# Doxorubicin Induced Nephrotoxicity: Protective Effect of Nicotinamide

**DOI:** 10.1155/2011/390238

**Published:** 2011-06-16

**Authors:** Sule Ayla, Ismail Seckin, Gamze Tanriverdi, Mujgan Cengiz, Mediha Eser, B. C. Soner, Gulperi Oktem

**Affiliations:** ^1^Suleymaniye Woman Health Hospital, 34122 Istanbul, Turkey; ^2^Department of Histology and Embryology, Cerrahpasa School of Medicine, Istanbul University, 34452 Istanbul, Turkey; ^3^Department of Medical Biology, Cerrahpasa School of Medicine, Istanbul University, 34452 Istanbul, Turkey; ^4^Department of Pharmacology, Meram School of Medicine, Selcuk University, 42080 Konya, Turkey; ^5^Department of Histology and Embryology, Ege University School of Medicine, 35100 Izmir, Turkey

## Abstract

*Introduction*. Nephrotoxicity is one of the important side effects of anthracycline antibiotics. The aim of this study was to investigate the effects of nicotinamide (NAD), an antioxidant agent, against nephrotoxicity induced by doxorubicin (DXR). *Methods*. The rats were divided into control, NAD alone, doxorubicin (20 mg/kg, i.p.) and DXR plus NAD (200 mg/kg, i.p.) groups. At the end of the 10th day, kidney tissues were removed for light microscopy and analysis. The level of tissues' catalase (CAT), glutathione (GSH), glutathione peroxidase (GPx), inducible nitric oxide (iNOS) and endothelial nitric oxide (eNOS) activities were determined. *Results*. The activities of CAT, GPx, and GSH were decreased, and Po was increased in renal tissue of doxorubicin group compared with other groups. The tissue of the doxorubicin group showed some histopathological changes such as glomerular vacuolization and degeneration, adhesion to Bowman's capsule and thickening and untidiness of tubular and glomerular capillary basement membranes. Histopathological examination showed that NAD prevented partly DXR-induced tubular and glomerular damage. *Conclusions*. Pretreatment with NAD protected renal tissues against DXR-induced nephrotoxicity. Preventive effects of NAD on these renal lesions may be via its antioxidant and anti-inflammatory action.

## 1. Introduction

Quinine-containing anthracycline antibiotic doxorubicin (DXR) has been used for the treatment of cancer since 1969. In spite of its high antitumor efficacy, DXR's use in chemotherapy has been largely limited due to its cardiac, renal, pulmonary, testicular, and hematological toxicities [1, 2]. DXR causes an imbalance between free oxygen radicals and antioxidants. The disturbance in oxidant-antioxidant systems which has been demonstrated with lipid peroxidation (LPO) and protein oxidation results with tissue injury [3]. Although the exact mechanism of DXR-induced nephrotoxicity remains unknown, it is believed that the toxicity may be mediated through free radical formation, iron-dependent oxidative damage of biological macromolecules, membrane LPO, and protein oxidation [4]. DXR-induced changes in the kidneys of rats include increased glomerular capillary permeability and tubular atrophy [5].

Nitric oxide (NO) is a free radical gas which acts as a cytoprotective or a cytotoxic agent. NO is generated by either endothelial nitric oxide synthase (eNOS) or inducible nitric oxide synthase (iNOS) [6]. Possible role of DXR in NOS metabolism occurs via direct or indirect stimulation of NO production, and this might be a consequence of increased free radical generation. Free radical production and/or NO release induced by DXR is entirely responsible for the DXR-induced toxicity [7]. Mitochondria has been defined as one of the targets in DXR-induced subcellular damage in the tissue. In addition, it has been shown that DXR could stimulate transmembranal arginine transport to provide increased substrate and activate NOS mediated NO-production [8].

Nicotinamide (NAD), a derivative of Vitamin B_3_, has been shown to exert a number of anti-inflammatory properties like iNOS inhibition suppression of both MHC class II and intracellular adhesion molecule expression on endothelial cells [9, 10]. It has been suggested that NAD has an ability to inhibit poly(ADP-ribose) polymerase (PARP) which has been defined as a nuclear DNA repair enzyme [11]. NAD has been shown to have antioxidant activity. Besides inhibiting protein oxidation and lipid peroxidation, it also inhibits reactive oxygen species-induced apoptosis [12]. The present study was therefore designed to investigate the effects of NAD on lipid peroxidation, antioxidant status, and iNOS activity in DXR-induced nephrotoxicity in rats. 

## 2. Materials and Methods

### 2.1. Animals and Experimental Protocol

Male Wistar-albino rats weighing 250 ± 30 g (mean-standard deviation) were used in the experiments. A standard diet and tap water were provided ad libitum. The experimental protocols were approved by the Cerrahpasa Medical School Animal Ethical Committee of Istanbul University. Control group was treated intraperitonealy with 0.9%, NaCl for 10 days (*n* = 6); NAD group was treated with i.p. NAD (Sigma Chemical Co., USA) alone for 10 days (200 mg/kg b.wt/day) (*n* = 8); DXR group was treated with single i.p. injection of DXR (20 mg/kg/b.wt) (*n* = 8) [13]; DXR + NAD group received DXR and NAD combination treatment (*n* = 8) in which NAD treatment was begun 1 day before DXR treatment.

### 2.2. Sample Collection and Biochemical Assay

At the 10th day of DXR treatment, the animals were anesthetized with pentobarbital (I.E. ULUGAY Istanbul, Turkey) (i.p. 6.5 mg/kg), the right kidney was rapidly excised and sectioned vertically into two pieces for microscopic examination and biochemical analysis. The renal tissue was stored at −70°C until biochemical analyses. Kidneys were thawed and homogenized (10%W/V) with 0.15 KCl at 4°C then centrifuged at 10,000 g for 1.5 h. The supernatant was used as the source of experimental product. Glutathione (GSH) assay was determined by the method of Beutler et al. [14] with 5.5 dithio bis nitrobenzoic acid as product. The catalase activity was performed by Aebi's method [15]. Glutathione peroxidase activity was measured by the method of Paglia and Valentine [16]. Protein oxidation was determined according to the technique which was reported by Levine et al. [17]. Protein levels were measured by Lowry method. All the measurements were done by schimadzu 1601 UV spectrophotometer. 

### 2.3. Light Microscopy

**Figure 1:**
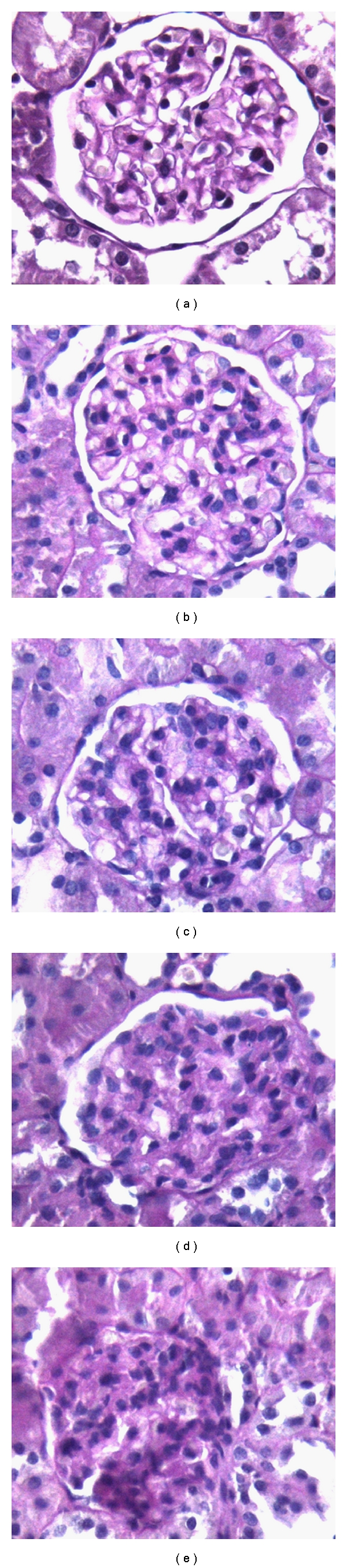
Mesangial matrix expansion (a) PAS ×40, grade 0; (b) PAS ×40, grade +1; (c) PAS ×40, grade +2; (d) PAS ×40, grade +3; (e) PAS ×40, grade +4.

The kidneys were sectioned and fixed in 10% formalin, dehydrated and embedded in paraffin. Tissues were sectioned at 5 *μ*m and stained with periodic acid Schiff (PAS). The histological slides of kidney were evaluated for semiquantitative analysis without knowledge of the treatment protocol, as described previously [18]. A semiquantitative score was developed to evaluate the degree of the damage. A minimum of 20 glomeruli (range 20 to 60) in each specimen was examined, and the severity of the lesion was graded from 0 to 4+ according to the percentage of glomerular involvement. Thus, a 1+ lesion represented an involvement of 25% of the glomerulus, while a 4+ lesion indicated that 100% of the glomerulus was involved. An injury score was then obtained by multiplying the degree of damage (0 to 4+) by the percentage of the glomeruli with the same degree of injury, that is, increase in mesangial matrix material or glomerulosclerosis. The extent of the injury for each individual tissue specimen was then obtained by the addition of these scores. For example, if 5 of 20 glomeruli had a lesion of 1+ and 5 of 20 had a lesion of 3+, the final injury score for that specimen would be (1 × 5/20) + (3 × 5/20) × 100 = 100. The injury score for individual tissue specimens derived by each investigator varied from 11% in the specimens with minimal changes (0 to 1+) up to 18% in specimens with more severe and widespread (2 to 4+) injury. The scores obtained by the two investigators were averaged (Figure 1). 

### 2.4. Electron Microscopy

The samples were dissected to 1 mm^3^ and fixed by 4% glutaraldehyde which is prepared by Soransen's phosphate buffer pH: 7.4 for 1 h. Samples were washed with phosphate buffer for 1 h and applied after fixation by Osmium tetroxide (OsO_4_) which is prepared by Milloning's buffer. The fixed samples were dehydrated in ethanol, embedded in Araldite, and cut into 500–700 Å. The cross-sections were then taken on copper grid and stained with uranyl acetate and Reynold's Leade Citrate. The coppers were investigated with Jeol JEM 1011. 

### 2.5. Tissue Processing and Immunohistochemistry

Tissue pieces were fixed in 4% paraformaldehyde (Sigma Chemical Co., St. Louis, Mo, USA) for 24 h at 4°C and processed for embedding in paraffin wax using routine protocols. 5 *μ*m thick coronal sections were cut using a microtome (Leica MR 2145); they were then dewaxed and rehydrated through a graded ethanol series using routine protocols. Sections were then washed with distilled water and phosphate buffered saline (PBS) for 10 minutes and then treated with 2% trypsin (Sigma chemical Co., St. Lois, Mo, USA) in 50 mM Tris buffer (pH 7.5), at 37°C, for 15 minutes. Sections were delineated with a Dako pen (Dako, Glostrup, Denmark) and incubated in a solution of 3% H_2_O_2_ for 15 minutes to inhibit endogenous peroxidase activity. Then, sections were incubated with primary antibodies directed against iNOS (1 : 100 dilution; Abcam, Cambridge, UK) and eNOS (1 : 1000 dilution; Abcam, Cambridge, UK) all for 18 h at 4°C in a humid chamber. Sections were then incubated with biotinylated secondary antibody and then with streptavidin conjugated to horseradish peroxidase (both from Zymed Histostain-plus-Peroxidase-kit, 85-9043, San Francisco, Calif, USA, prepared according to manufacturer's instructions) for 30 min each. Finally, sections were incubated with diaminobenzidine (DAB) (from DeadEnd Colorimetric TUNEL system, Promega, Madison, USA, prepared according to manufacturer's instructions) for 5 min to reveal immunolabelling. All dilutions and thorough washes between stages were performed using PBS. Sections were counterstained with Mayer's hematoxylin (Zymed Laboratories, USA). After washing with tap water, sections were dehydrated through a graded ethanol series, cleared in xylene, and mounted with Entellan (Merck). Negative control samples were processed as described above except that primary antibodies were omitted and replaced with PBS alone. Positive controls were represented by sections of a neuroblastoma specimen known to be positive for the markers of interest.

### 2.6. Evaluation of Immunohistochemical Sections

Immunohistochemistry was evaluated semiquantitatively (Olympus BX-51 and Olympus C-5050 digital camera) using an additive immunoreactive score reflecting signal intensity, that is 0-negative, 1-weak, 2-intermediate, and 3-strong, and the number of immunopositive cells, that is, 0-no positive cells, 1-less than 10% positive cells, 2–10% to 50%, and 3-greater than 50%. 2 scores were added. Measurement was performed by two independent researchers blind to the drug administration groups.

### 2.7. Statistical Analysis

Data were analyzed by using a commercially available statistics software package (SPSS for Windows v.10.0, Chicago, USA). Since measurement values did not display homogeneous distribution, Kruskal-Wallis variance analysis test has been used to evaluate the meaning of the difference among the groups. The variance analysis results which were found meaningful were cross-checked with Mann-Whitney *U*-test. Results were presented as mean ± S.E.M. *P* values <0.05 were regarded as statistically significant. 

## 3. Results

### 3.1. CAT, GPx, and GSH Analysis of Kidney Tissue

The biochemical results of renal tissue are illustrated in Table 1. The renal CAT, GPx, and GSH activities were significantly lower in DXR group then the other groups (*P* < 0.001). The levels of Po in renal tissue were significantly increased in DXR group when compared with other groups (*P* < 0.001). 

### 3.2. Effect of NAD in DXR-Induced Toxicity by Light Microscopic Evaluation

**Figure 2:**
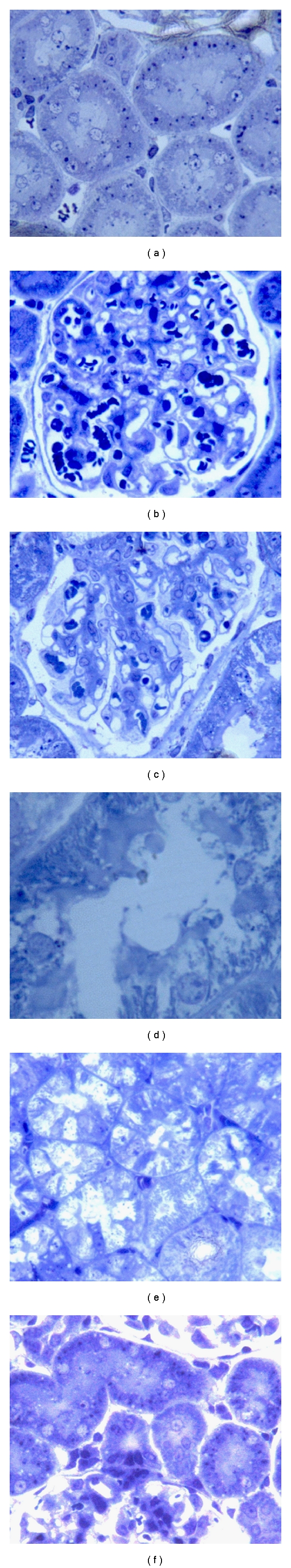
Control and nicotinamide (NAD), (a) and (b) toluidine blue ×40; doxorubicin (DXR); (c) toluidine blue ×40; (d) toluidine blue ×100; (e) toluidine blue ×40; DXR + NAD; (f) toluidine blue ×40.

**Figure 3:**
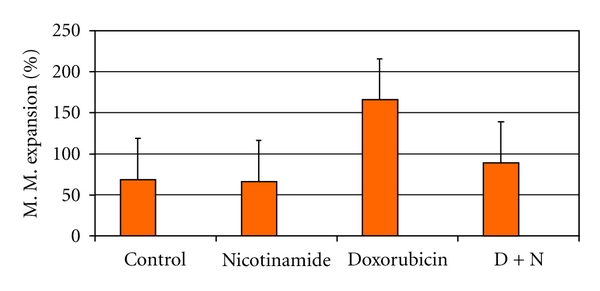
Mesangial matrix expansion % tissue in the kidney glomeruli.

There was no abnormal findings for the kidney of both control and NAD groups in the light microscopic examination (Figures 2(a) and 2(b)). Degenerative changes were observed in the renal glomeruli and tubules of only DXR group. The urinary spaces and capillaries were dilated, and the flat epithelial cells of the parietal layer of Bowman's membrane could be discerned mostly as cuboidal or round in shape. In the proximal tubules, vacuolization was observed in the endothelial cell cytoplasm, for the most part, degenerated, and microvillus is lost (Figures 2(c), 2(d), and 2(e)). Treatment with NAD resulted in almost normal tubules and glomeruli in the light microscopic examination (Figure 2(f)). The graded histological changes (Mesangial matrix expansion) are summarized in Figure 3. 

### 3.3. Ultrastructural Changes of Kidney Cells

**Figure 4:**
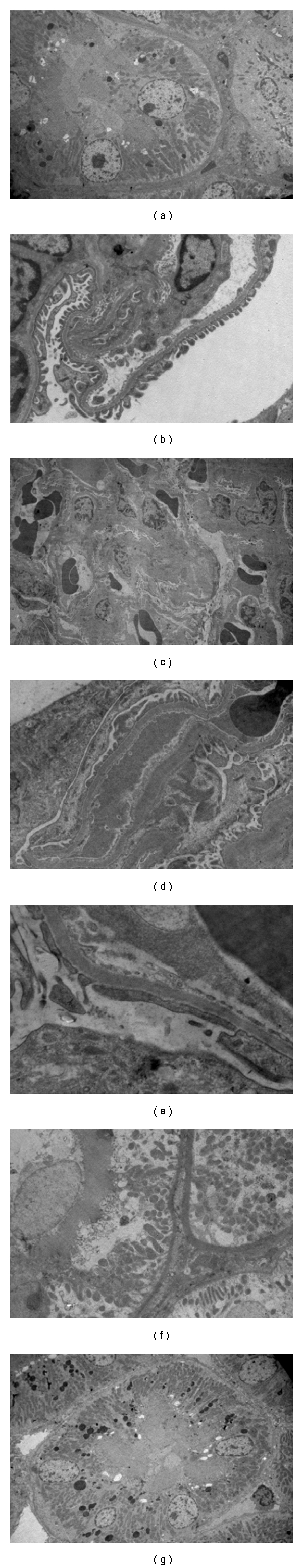
(a) and (b) Control ×3000 and nicotinamide (NAD) ×10 K; doxorubicin (DXR), (c) ×3000, (d) ×10 k, (e) ×30 K, (f) ×5000, DXR + NAD, (g) ×500.

Structure of kidneys in control and NAD groups was evaluated in the electron microscopy (Figures 4(a) and 4(b)). Increased mesangial matrix (Figure 4(c)), thickening, and untidiness of glomerular capillary basement membranes were determined in DXR groups (Figure 4(d)). In the glomerular area, the cellular integrity of podocytes was compromised, and the cytoplasmic foot processes had been withdrawn and adhered to each other (Figure 4(e)). Degenerative changes were found in the proximal tubules, and spaces were observed in the cytoplasm, forming wide, vacant regions between the nuclear and basal membranes (Figure 4(f)). In DXR + NAD group, the cellular structure was better preserved when compared with DXR group, and the structure of tubules was better preserved when compared with DXR group. Treatment with DXR + NAD resulted in almost normal tubules (Figure 4(g)).

### 3.4. Expression of Inducible and Endothelial Nitric Oxide Synthase

**Figure 5:**
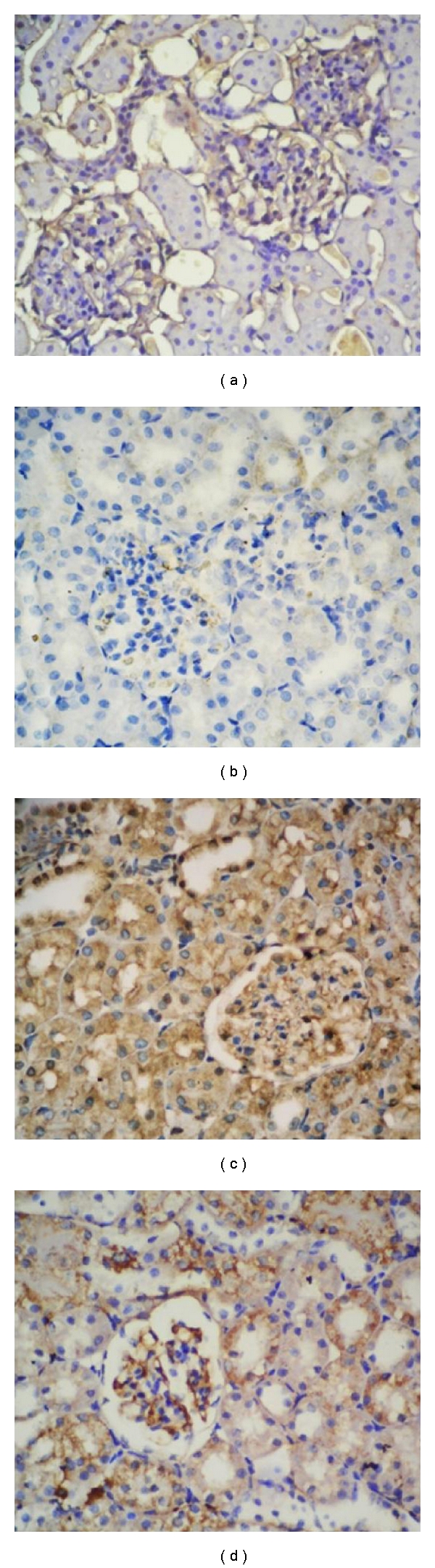
(a) and (b) iNOS control and nicotinamide (NAD), (c) Doxorubicin (DXR), (d) DXR + NAD.

**Figure 6:**
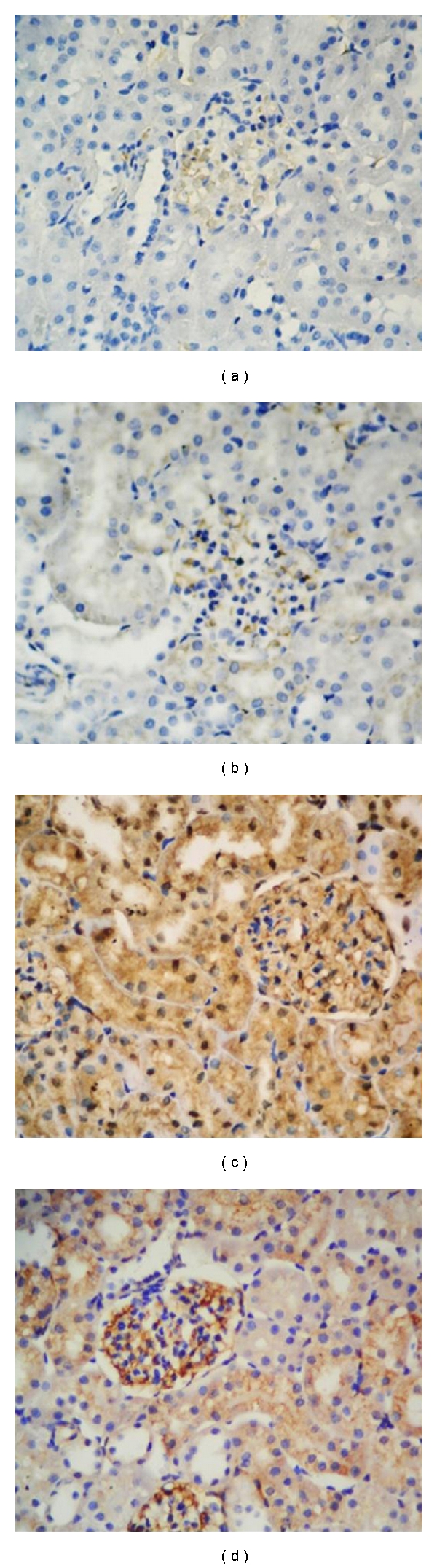
(a) and (b) eNOS control and nicotinamide (NAD), (c) doxorubicin (DXR), (d) DXR + NAD.

iNOS and eNOS immunoreactivities were investigated in the sections. Immunohistochemical analyses demonstrate that iNOS (Figure 5(a)) and eNOS (Figure 6(a)) expression was weak in the control group. After DXR application, both iNOS (Figure 5(c)) and eNOS (Figure 6(c)) immunoreactivities were increased significantly in kidney tissue. In DXR group, kidney sections showed increased expression of eNOS in interstitial, endothelial, and macula densa cells, while decreased expression was obtained in NAD-treated group (Figures 5(b)–6(b)). Immunohistochemical reaction was strong in DXR and weak in NAD group when compared with control group. Combined application of DXR and NAD showed intermediate iNOS (Figure 5(d)) and eNOS (Figure 6(d)) expression in kidney tissue. In summary, iNOS and eNOS immunohistochemical analysis demonstrated markedly decreased effect of NAD in DXR-induced kidney toxicity. DXR-induced toxicity showed an increased iNOS and eNOS immunohistochemical analysis results, and this effect was markedly decreased with NAD treatment.

## 4. Conclusion

In the present study, our first goal was to demonstrate the intense correlation between DXR-related iNOS and eNOS immunoreactivity in tissue damage and the protective effects of NAD with decreased iNOS and eNOS immunoreactivity. Moreover, we showed DXR-induced tissue injury in the kidney, and the damage was demonstrated by microscopic and biochemical evaluations. 

Anticancer therapy usually demolishes the physiological homoeostasis and affects multiple organs during treatment process. Effective anticancer therapy with anthracyclines is limited because of its toxicity to various organs including kidneys [19, 20]. The toxicity has been attributed to radical formation and oxidant injury. Nephrotoxic action of DXR is also considered to be via drug-induced free radical generation [21, 22]. The formation of free radicals as well as an increase in response to DXR treatment has already been documented. The disturbance in oxidant-antioxidant systems results in tissue injury that is demonstrated with protein oxidation in tissue and protein oxidation in renal tissue, is recognized as one of the possible biochemical mechanisms of DXR-induced nephrotoxicity [3], and we have found that DXR treatment raised Po (protein oxidation product) in rat kidney.

NOS may be responsible for the reductive activation of DXR to its free radical semiquinone form and the subsequent oxygen radical-mediated cellular damage [23]. Our results showed the elevated immunoactivation of both iNOS and eNOS after DXR treatment. In DXR group, ultrastructural changes such as cellular damage and glomerular and tubular degeneration were determined. 

GSH plays an important role in the detoxification of xenobiotic compounds and in the antioxidation of reactive oxygen species and free radicals. Low levels of GSH were observed in oxidative stress. This observation supports our finding in which we have observed a decline in GSH levels with an increase in oxidative stress as evidenced by increased LPO [24]. A decrease in the activity of CAT was observed with DXR administration [25]. The above finding corroborates with our results where we have observed a reduction in the CAT activity.

Deman et al. [22] demonstrated reduced glutathione concentration in the renal cortex supports the idea of free radical involvement in nephrotoxicity of DXR. In the present study, we showed reduced glutathione in the renal tissue. It was shown by Yagmurca et al. [13] that 20 mg/kg single dose of DXR resulted in renal LPO at the 10th day of DXR injection in rats. We also demonstrated that CAT, GSH, and GPx activities were decreased 10 days after DXR treatment. Yagmurca et al. demonstrated that glomerular sclerosis was seen 10 days after DXR injection in rats. Also, it was shown that there was thickening of capillary basement membrane in the DXR group. Urinary spaces and capillaries were dilated, and the flat epithelial cells of the parietal layer of Bowman's membrane could be discerned mostly as cuboidal or round in shape. In the proximal tubules, vacuolization was observed in the endothelial cell cytoplasm, for the most part, degenerated, and microvillus is lost (Figure 2(d)). Studies carried out by Fajarda et al. [26] and Strenberg et al. [27] showed that cytoplasmic foot processes of podocytes were damaged with DXR. Identical results were also found in our study. Vacuoles were observed in the cytoplasm between the nuclei and cellular membranes of the tubules.

 Another radical formatting mechanism in such an experimental protocol might be NO producing system. The high production of NO results in peroxynitrite formation via NO reacting with superoxide anion. Peroxynitrite is a potent and aggressive cellular oxidant and causes the formation of 3-nitro-L-tyrosine [28]. iNOS is involved in the inflammatory process. It was also shown in recent years that high NO production is involved in DXR toxicity [29, 30]. The present study demonstrated that NO production was increased in renal tissue of DXR-treated rats. As control and NAD groups' area that has been dyed overly, it might also be related to the inflammatory answer of the tissue against DXR. 

DXR has widely been used in many countries for hematological malignancies. However, the toxic effect on the kidneys and consequently acute renal failure producing effect of DXR is a limiting factor of its usefulness. Therefore, novel therapeutic agents with improved efficacy seem to be considerable for clinical approach. NAD has been shown to possess anti-inflammatory, anticancer, and antioxidant properties [31]. Previous studies have demonstrated that NAD exhibits antioxidant properties against oxidant conditions that cause tissue injury and may prevent carbon tetrachloride-induced liver fibrosis in rat via antioxidant mechanism [31]. The present study indicated that NAD treatment caused decreased LPO and protein oxidation in the kidney tissue after DXR administration. Also, NAD prevented the histopathological changes occurred due to DXR toxicity in rat kidney in this study.

In conclusion, the present study demonstrates that 20 mg/kg single injection of DXR to the Wistar-Albino rats caused renal injury including glomerular and tubular lesion 10 days after the DXR injection. Furthermore, this study revealed that pretreatment with NAD protected renal tissues against DXR-induced nephrotoxicity. Preventive effects of NAD on these renal lesions may be via its antioxidant and anti-inflammatory action. Although the exact mechanisms remain to be clarified, NAD could be an effective course of therapy to enhance therapeutic efficacy and to lessen DXR toxicity in clinical chemotherapy. 

## Figures and Tables

**Table 1:** The activities of catalase (CAT), glutathione peroxidase (GPx), glutathione (GSH), and protein oxidation (Po) levels in renal tissue of control (*n* = 6), NAD (*n* = 8), DXR (*n* = 8), and DXR plus NAD groups (*n* = 8). *P = 0.001; control versus NAD, DXR, and N + D, **P = 0.105; control versus N + D.

	Markers			
Groups	GSH (*μ*moL/mg protein)	GPx (*μ*moL/*μ*g)	CAT (*μ*moL/*μ*g)	Protein oxidation (nmoL/*μ*g)
Control	5.32 ± 1.39	1.73 ± 0.38	9.16 ± 4.16	0.35 ± 0.04
NAD	4.85 ± 1.15*	0.60 ± 0.11*	4.61 ± 0.36*	0.15 ± 0.02*
DXR	2.22 ± 1.06*	0.41 ± 0.17*	3.84 ± 0.99*	4.32 ± 0.20*
DXR + NAD	4.90 ± 0.9**	1.48 ± 0.15*	20.01 ± 7.40*	0.68 ± 0.11*

## Abbreviations

NAD: Nicotinamide
DXR: Doxorubicin
GSH: Glutathione
CAT: Catalase
GPx: Glutathione peroxidase
Po: Protein oxidation
iNOS: Inducible nitric oxide synthase
eNOS: Endothelial nitric oxide synthase
PAS: Periodic acid-Schiff
LPO: Lipid peroxidation.
